# A digital innovation typology: Navigating the complexity of emerging technologies to negotiate health systems research with young people

**DOI:** 10.1177/20552076231212286

**Published:** 2023-11-07

**Authors:** Teresa Swist, Philippa Collin, John Lewis, Sharon Medlow, Ian Williams, Cristyn Davies, Katharine Steinbeck

**Affiliations:** 1 Institute for Culture and Society, Young and Resilient Research Centre, 89381 Western Sydney University, Penrith, NSW, Australia; 2 Education Futures Studio, 153403 Sydney School of Education and Social Work, University of Sydney, Camperdown, NSW, Australia; 3 Wellbeing Health & Youth Commission, Sydney, NSW, Australia; 4 Speciality of Child and Adolescent Health, 522555 Faculty of Medicine and Health, Sydney Medical School, 153403 The University of Sydney, Sydney, NSW, Australia; 5 Academic Department of Adolescent Medicine, The Children's Hospital Westmead, Westmead, NSW, Australia; 6 Medicine, Dentistry and Health Sciences, 85084 The University of Melbourne, Melbourne, VIC, Australia; 7 Specialty of Child and Adolescent Health, 522555 Faculty of Medicine and Health, 89381 University of Sydney, Children's Hospital Westmead Clinical School, Westmead, NSW, Australia

**Keywords:** Digital health < general, health communications < general, internet < general, technology < general, qualitative < studies, media, integrated knowledge translation, innovation, emerging technologies, typology

## Abstract

**Objective:**

This study aims to explore young people's perspectives of emerging technologies and health systems research in an adolescent health community of practice.

**Methods:**

The context of this integrated knowledge translation study is the Wellbeing Health & Youth Centre of Research Excellence in Adolescent Health. A theory-building, non-systematic review was conducted to examine the concepts and interrelationships of emerging technologies associated with digital innovation and health systems. This typology informed the design of an online workshop with young people to explore their views, concerns, and ideas about health systems research.

**Results:**

A digital innovation typology was identified to differentiate and explain emerging technology concepts and interrelationships that can be applied to the health systems context. Aligned with this typology, youth perspectives about digital health challenges and opportunities were identified to support future research, policy, and practice.

**Conclusion:**

The integrated findings from this study can assist the navigation of complex emerging technologies, and the negotiation of equitable health systems research, between youth and adult stakeholders. Further, with these typology-related resources, mutual learning and the public involvement of young people in health systems research and priority setting agendas can be supported.

## Introduction

Understanding the social implications of rapid technological change is a global concern for all age groups and sectors of society. An increasingly sophisticated array of everyday and emerging technologies has the potential to transform the future of health systems.^1,2^ Chat-bots, or targeted advertisements on mobile phones or computer rely on *Artificial Intelligence* (AI), and algorithmic training by data to perform specific tasks to respond to questions or recommend products or services, as the *Internet-of-Things* (IoT). Data collected via online searches and fitness trackers contribute to a growing volume and velocity of data, termed *Big Data*. These technologies change perceptions of self and the body, present new ways of participation in health (online communities, help-seeking) and produce shifts in the production of health-related knowledge and innovation, including data sharing.^3,4^ To address these complexities, the study of ‘digital health’ requires new interdisciplinary, theoretical, and engaged approaches to understand and explain the complex, health-related phenomena of people's everyday lives.^5^ In particular, a focus upon ‘responsible research and innovation’ across health systems brings a focus upon envisioning ‘what types of innovations health systems need and how they should be produced and bought to market in order to support equitable and sustainable health systems around the world’.^6^

Various organisations have strategic visions for future health and technology agendas: a vision for ‘evolving health and care’ to be safe, seamless, and secure^7^; incentivising technology development to reduce negative environmental impacts^8^; and a human rights approach to the development and use of new and emerging technologies.^9^ The production lifecycle of emerging technologies and their applications are increasingly complex for diverse population groups, prompting investigation on the needs of citizens across all stages of life in the development of AI, IoT, and Big Data technologies.^10–12^ Such investigation enhances public understanding, and could create ‘informed citizens’ and minimise exploitation and discrimination.^13^

Public involvement in research is about tailored ‘two-way learning’.^14^ How people talk about, and understand, these emerging technologies and interrelationships, therefore requires shared understandings between people of diverse ages, expertise, and experiences.^15,16^ In doing so, conversations about how data-driven technologies operate in everyday circumstances can move beyond cycles of hype and fear to inform more inclusive, appropriate and effective design of such technologies.^17^ Attention to a ‘cultural contexts’ health approach helps to foreground the importance of such shared and everyday learning; in particular, ‘the diverse and shifting parameters within with decisions and actions unfold’ that can potentially enhance practices and innovations.^18^

Historically, innovations in health systems are slow-paced and focused on products, services, or care pathways in isolation from one another.^19^ In the twenty-first century, innovation in reality involves many stakeholder interactions, organisational and policy mechanisms, and cross-sectoral interdependencies which occur together, systemically, and over time.^20^ The evolution of digital health is therefore shaped by citizens, consumers, multinational technology companies, other industries, and health professionals, data capture and usage, and broader understandings of health beyond clinical settings.^21^ To simplify this health system complexity, a process of ‘cultural intelligence’ is essential to identify and understand the interrelated forces driving rapid social and technological change.^22^ Also, attention to the contextuality of data, or ‘thick data’,^23,24^ associated with emerging technologies can help surface multi-layered motivations and practices. To inform contextual action or change, collaborative inquires utilising participatory research can co-construct partnerships between researchers and others towards this common purpose.^25^

Examples of advancing digital technologies include: ‘smart’ connected devices, the Internet of Things, machine learning, advanced computing, big data analytics and robotics.^26^ These emerging technologies are truly novel, have the potential to significantly change practices, carry uncertain ethical and social implications, and unpredictability of future directions.^27,28^ Large consumer technology companies are at the forefront of this research and development. Their foray into health research has benefits and drawbacks, as well as significant implications for both public health institutions and public trust about data collection and use.^29^ The way that health data are collected and deployed via consumer mobile devices is increasingly opaque and removed from individual control.^30^ Research stresses the need to focus upon ‘digital traces, algorithmic surveillance, and data-based discrimination’,^31^^, p.144^ as everyday technologies increasingly reproduce, rather than address, health inequities - including for young people. In particular, insights from information systems research and contexts of use can help to open up the ‘black-box’^32^ of digital technologies to ‘learn how and why technologies are designed and used in particular ways’,^33^^, p.3^.

Existing minority world research tends to focus on young people's use of health services, rather than on the emerging technologies and contexts that inform health systems innovation. Yet young people are highly likely to be online and using technologies for health.^34^ Global studies of young people's perspectives on life in the digital age in relation to health and well-being point to ‘limited evidence about the impacts of digital technologies on children and young people's health and happiness’,^35^^, p.78^. Future IoT research should examine implications of IoT for the healthcare sector and consider benefits and designs for diverse users, together with potential impacts upon existing inequalities and new harms that may affect children and young people.^36^ Youth engagement must be part of a collective, collaborative effort to address key priority areas, including the need to explore how technology innovations might boost access to services and address data gaps to improve health equity.^37,38^

The potential for leveraging new technologies for young people's healthcare thus relies upon not only access to the internet and devices, but also the resolution of ethical and legal considerations associated with digital health data. As examples, the application of AI in youth health may be shaped by diverse issues, such as: data bias with discriminatory outcomes for minorities, surveillance capitalism by tracking and monetisation of consumer data, and the implication of parental sharing of information about their children in social networks with negative short and longer-term implications.^39^ Another consideration is while technology brings opportunities for marginalised young people to access health services in new ways, access is unlikely without enhanced consumer health system knowledge and navigation support.^40,41^ Ultimately, poor access to the internet creates a digital divide exacerbating pre-existing socio-economic inequalities across all ages, a divide globally amplified during the COVID pandemic.^42^ In sum, there is a pressing need to overhaul the design and development of emerging technologies for health systems research in a way that connects with young people's lived experiences, views and needs.

### Aims

This paper aims to clarify concepts and explain relationships associated with emerging technologies, focussing on the implications for health systems research using the perspectives of young people. Our goal was to identify what conceptual and practical tools could support two-way learning between youth and adult stakeholders in health systems research and health service innovation. The study has three objectives: first, to review the literature about the interrelationship between emerging technologies and health systems, with specific consideration of the implications for research with young people; second, to investigate young people's perspectives of emerging technology and health systems; and, third, to inform future research agenda-setting. The identified outcomes were the creation of a *Digital Innovation Typology*, young people's perspectives aligned with the typology, and a synthesis of study insights to inform future research, policy, and practice agenda-setting.

## Methods

### Study approach and context: An integrated knowledge translation study

An integrated knowledge translation (IKT) approach prioritises a collaborative process between researchers and knowledge users to address a particular issue, which seeks to build trust, knowledge production, and shared visions of how research can and should be conducted.^43–45^ In 2018, the WH&Y NHMRC Centre of Research Excellence in Adolescent Health^46^ established a process for integrating youth engagement across health research and its translation. A co-produced framework specified that particular values, such as diversity and inclusion, can be explored and demonstrated by stakeholders ‘producing a common language and meaningful technologies, so that communications and resources are relevant to young people's everyday lives’,^47^^, p.9^. From the start of this initiative, young people have informed and guided research, and shaped its present form: the Wellbeing Health & Youth Commission with members regularly participating workshops to inform research, policy and practice.^48^ While there are global frameworks for planning, developing, and implementing solutions with and for young people which provide high-level guidance,^49^ there is recognised value in co-creating frameworks with specific communities and stakeholders for more context-specific practice.^50^

### Phase 1 method: Theory-building review

A theory-building, non-systematic literature review conducted by the lead author commenced this phase. Such reviews vary in style, yet their common objective is to be informative and help researchers understand the topic by integrating literature from diverse sources, so as to better comprehend a new phenomenon, provoke novel thought, identify trends, plus guide strategy and decision-making.^51,52^ The transdisciplinary purpose of this review was to iteratively select and synthesise pertinent academic and grey literature for illustrating interactions within complex systems.^53^ The reflexive, theory-building strategy was guided by science and technology studies literature, alongside search terms such as digital health, innovation, data, digital platforms, research, youth, artificial intelligence, internet of things. All articles selected were in written in English and published between 1987 and 2023. In stage one, the concept of ‘technological convergence’^54^ was utilised to frame how innovative practices emerge from the interplay of existing and emerging technologies. The second stage applied the analytical lens of ‘infrastructure’^55^ to illustrate the underlying, systemic interrelationship between technologies, stakeholders, and knowledge production. A *Digital Innovation Typology* was the main outcome of Phase 1, accompanied by a *Digital Health Dictionary*. A typology is best described as a classification system that offers a way to segment and order discrete categories for a particular field of knowledge^56^ The value of typology theory-building is to reduce the complexity of a phenomenon by articulating key concepts and associated interrelationships of the examined phenomenon.^57^

### Phase 2 method: Youth perspectives on digital health

Study participants were recruited from the Wellbeing Health & Youth Commission (WH&Y-C), an integral part of the Centre of Research Excellence in Adolescent Health which supported this research. Prospective study participants were informed about the online workshops via the group's closed Facebook page, and offered a gift voucher ($100AUD) for their participation. This workshop activity was conducted with eleven participants from the WH&Y-C as part of a regular two-hour workshop (conducted online via Zoom due to COVID-19 restrictions). The purpose of this activity was to use the *Digital Innovation Typology* to communicate emerging technology concepts and interrelationships so as to frame the workshop discussion and support two-way learning between researchers and commissioners. A workshop pre-reading, *Incredible Tales from the Near Future: Health in your Hands*^58^ was shared with the group. The comic's narrative focuses on a young person who, via her smartphone, is continually nudged by a chatbot that can monitor health, connect with services, and update data and information. Workshop discussion questions developed by the lead author focused on emerging technology implications for health-related monitoring, information exchange, and knowledge production (including role of smartphones, chatbots, data exchange and donation, automation, systemic approaches to health and wellbeing). Three scribes (co-author PC and two doctoral students) documented the discussion insights, which were analysed by the lead author to align with the review findings. This study was approved by the Western Sydney University Human Research Ethics Committee (Approval number: H11940) and written informed consent was obtained from participants prior to study initiation.

## Results

### Phase 1 results: A digital innovation typology to differentiate and explain emerging technology concepts and interrelationships that can be applied to the health context

**Diagram 1.**
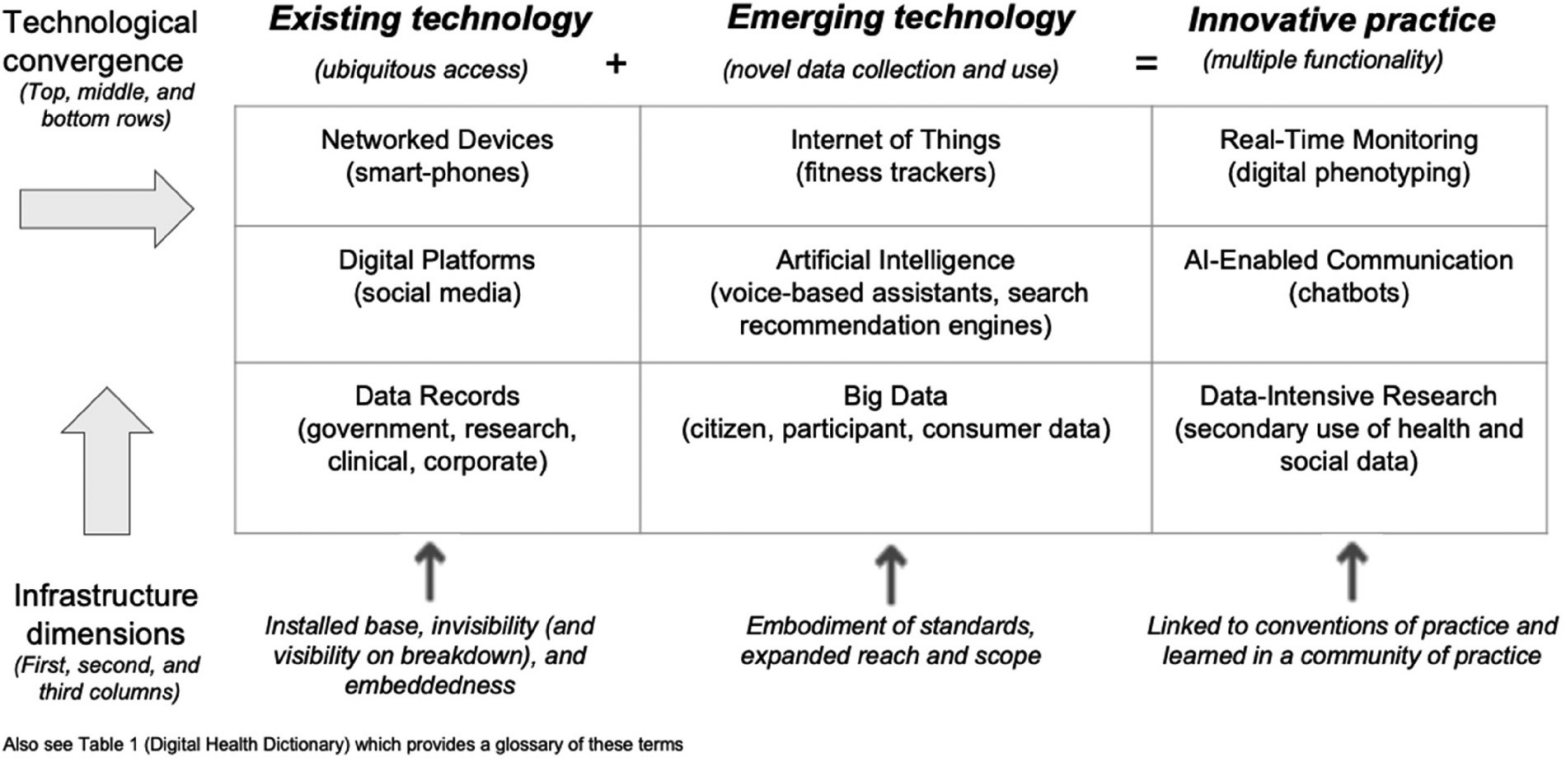
A digital innovation typology.

The key concepts central to understanding emerging technologies from a systemic perspective are represented as an individual cell, or variant (with examples), as part of the Digital Innovation Typology (Diagram 1). These key concepts are defined in the Digital Health Dictionary (Table 1).

*i) Technological convergence findings*

**Table 1.** Digital health dictionary.

Artificial Intelligence (AI)*.*	*Computing systems designed to perceive their environment and perform actions aligned with a given goal, which operate in relation to **digital platforms** and **networked devices***.
AI-Enabled Communication*.*	*The automated, or semi-automated, communicative practices between humans and machines which operate due to the convergence of **digital platforms** and **artificial intelligence***.
Data Records*.*	*The organisational software systems (used for individual, government, clinical, and business use) which standardise and classify data about people's identities (as citizens, patients, and consumers).*
Big Data.	*The management of diverse, often multimodal, **data records** about human and environmental behaviour according to a range of public health, commercial, or academic interests.*
Data-Intensive Research*.*	*The organisational research practices which evolve in relation to the convergence of **data records** and **big data**.*
Digital Platforms*.*	*Software that connects online tools and enables input, interactivity, and two-way communication via **networked devices** to manage communicative practices across multiple settings*.
*Internet-of-Things (IoT)*	*Interconnected **networked devices** with sensors and actuators which operate without involvement, or with limited involvement, of humans (smartphones, fitness trackers, or fixed sensors embedded in homes, healthcare institutions, urban settings)*.
Networked Devices (mobile, wireless, cabled)	*Mobile, wireless, or cabled **networked devices** reliant on connection to a telecommunications network (current 5G network, and 6G network under development), which operate a range of computer-based mechanisms (smartphones, smartwatches, computers, robots)*.
Real-Time Monitoring*.*	*The continuous monitoring practices which can occur anytime and anywhere in relation to an individual, group, or event, due to the convergence of **networked devices** and **Internet-of-Things***.

Innovative practice is demonstrated by ‘technological convergence’, whereby two or more independent technologies integrate to produce a new type of practice, or activity.^54^ For instance: *ubiquitous access* due to networked devices, platforms and records (*existing technology*); plus *novel data collection and use* due to the diversification of data capture, formats and algorithmic techniques (*emerging technology*); combine to equal, or produce, *multiple functionality* (*innovative practice*) when applied to specific contexts.

The top row of the typology displays the convergence of Networked Devices and the Internet of Things to produce Real-Time Monitoring. An example of this is ‘digital phenotyping’: the potential to continually assess behaviour and mood with the *ubiquitous access* of existing networked devices, due to integration with emerging IoT that can capture *novel data collection and use*, such as physiological and biometric data. Digital phenotyping thus catalyses the *multiple functionality* and innovative practice of real-time monitoring. An example of challenges identified within digital phenotyping studies in the adolescent mental health field include: consent and privacy concerns for both an individual and the community regarding the type of information collected and the development of the field as a whole; and, accountability, regulation and liability issues - such as commercial breaches.^59^ Mental health is a specific application of digital phenotyping. However, digital phenotyping has range of ethical, legal, and social implications more broadly for healthcare and health policy.^60^

The middle row of the typology displays the convergence of Digital Platforms and AI to produce AI-Enabled Communication. An example of this is ‘chatbots’: an AI-enabled agent embedded in the *ubiquitous access* of existing digital platforms and integrated with the *novel data collection and use* of an organisation's AI software. This combination of technologies catalyses the *multiple functionality* and innovative practice of AI-enabled communication, allowing responses to questions by simulating human-like conversation. The potential convenience of health chatbots for young people could encourage more young people to seek support. However, the limited oversight of and lack of ongoing evaluation of chatbots means that harms could occur undetected. Research is needed on how best to respect users’ privacy, ensure users’ safety, and provide greater transparency about what chatbots can realistically offer.^61^

The bottom row of the typology displays the convergence of Data Records and Big Data to produce Data-Intensive Research. An example of this is My Health Record (MHR) in Australia.^62^ This is an individual digital health record linked to the *ubiquitous access* of digitally-stored clinical data records. The potential to integrate this form of health record with *novel data collection and use* of emerging Big Data has already caused controversy with the UK National Health Service's COVID-19 data platform.^63^ The innovative practice of data-intensive research stems from the *multiple functionality* related to secondary use of health and social data. The challenges associated with integrating the secondary use of administrative data sets with other data sets collected by devices include technical limitations, such as interoperability, and ethical issues such as privacy and consent.^64^

*ii) Infrastructure findings*

Diagram 1 can also be read vertically according to various aspects of infrastructure which is both social *and* technological. Star & Ruhleder's^55^ dimensions of infrastructure were deployed as an analytical lens to explain how these features relate to the concepts and technological convergence in the *Digital Innovation Typology*.

The first column comprises the following infrastructure dimensions - *installed base, invisibility (and visibility on breakdown),* plus *embeddedness* in everyday life - which enable the ubiquitous access associated with technological convergence. The *installed base* of digital health is comprised of a group of existing technologies (networked devices, digital platforms, and data records) upon which all emerging technologies are built. These foundational technologies are characterised by *invisibility (and visibility on breakdown)*. Invisibility includes the ways in which existing technologies blend into the background of supporting everyday tasks, and, conversely, become very visible upon breakdown of these technologies. Battery depletion, interrupted wi-fi access, hacking of legacy operating systems, data-breaches, power failures – or intense public scrutiny with failure to protect privacy - are examples of the range of technical and trust-related breakdowns. The *embeddedness* of existing technologies across everyday life and information sharing, relates to the extent to which these become mainstreamed across society with ubiquitous use. We only notice their absence or limitations, when there is a breakdown, glitch, or controversy. These infrastructure dimensions underpin the ‘Internet of Health’, where anyone linked to a device, digital platform, or database record is subject to a system of continuous health-related monitoring, diagnosis and prediction.^65^

The second column of the typology comprises *embodiment of standards* and *expanded reach and scope,* which enable novel data collection associated with emerging technological convergence. Digital data collection diversifies with the *embodiment of standards* that enable these technologies to easily ‘plug into’ other technology and device infrastructures. Emerging technologies are integrated with existing technologies (the installed base) through technical standardisation. For example, the IoT operates via your smart-phone, AI-recommended advertisements operate via your favourite social media digital platforms, and Big Data operates via expanded data records. This produces *expanded reach and scope* of what infrastructures support, often beyond a single site of practice, or one point in time. For instance, networked devices like sensors (that produce and exchange data) and actuators (which control a mechanical action) with minimal or no involvement from humans.

The third column of the typology introduces *links with conventions of practice* and *learned as part of a membership within a community* which shape the multiple functionalities produced with technological convergence. Innovative practices of real-time monitoring, AI-enabled communication, and data-intensive research are learned and become accepted as part of a membership within a community of stakeholders with shared interests. Infrastructure *links with conventions of practice* (such as existing organisational routines) and is thus *learned as part of a membership* within a particular community of practice (which is interdependent with organisational resources and interests). This means the advancement and direction of real-time monitoring, AI-enabled communication, and data-intensive research all rely upon being learned, and shaped, by different stakeholders: either as individuals (as a family or community member, patient, clinician, researcher, other professional) or as groups (defined by age, diagnosis, geography, or investment strategy).

### Phase 2 results: Alignment of young people's perspectives with typology concepts

*i) Networked devices + IoT = Real-time monitoring*

Commissioners reported that a major advantage of using ubiquitous *networked devices* for health and well-being was convenience, especially via mobile phones. The design of online environments or interfaces which nudge people's behaviour was viewed as potentially advantageous in terms of looking out for them if they were neglecting themselves. Key advantages associated with digital nudges included increasing awareness of bad habits, or that the prompts offered could promote mindfulness or be useful for tracking weight and calorie intake. However, continual nudging was viewed as potentially negative, specifically in terms of influencing young people's decision-making, attitudes and behaviours in their everyday lives (such as for young people with issues which might be exacerbated by potentially negative nudging). Concerns about privacy and data sharing were also raised, including the need for clear information about how to opt out and/or delete data. Commissioners noted that ongoing sensing and tracking associated with the large-scale generation of health and social data was ominous and requires trust-building. They felt that the contexts in which health information is collected and made accessible by networked devices should be made more transparent to young people; this was viewed as significant, because the amount of information to which a young person may consent is context specific.

Commissioners considered the *Internet-of-Things (IoT*) in relation to fitness trackers (as one concrete example of consumer health technology amid IoT innovation more broadly). They discussed how fitness tracker data did not always correctly align with a young person's broader context or lived experience, or could be manipulated to accidentally, or purposefully, generate erroneous data. For example, making it seem like a young person is exercising, when they are just taking a hot shower. Young people shared that shaking an arm vigorously to push steps up could be used to trick the tracker (and parents) into believing that a young person's exercise regimen is on track. In addition, while the social aspect of sharing activity tracking updates with peers may be motivational to some, the competitive aspect can also impact upon identity, self-esteem and reputation. WH&Y Commissioners expressed that fitness tracking, such as automated *real-time monitoring* and feedback, was changing their views about health-related roles and practices in their everyday lives. Significantly, the value of seeing a health professional in-person is that they may identify concerns not apparent via phone, video, or more diverse types of data, such as fitness tracking. Furthermore, self-monitoring and reporting can add the pressure of ‘playing the doctor’ role, meaning that situations could arise where people overlook symptoms (due to information overload), or lie about or fake symptoms. In-person check-ups therefore offer the opportunity to explore or assess a broader range of symptoms and the context or concerns of young people. Telemonitoring from a distance such as phone counselling or telehealth, was considered a positive option for some purposes, but Commissioners viewed some check-ups as needing an in-person presence. While the promises of emerging technologies in health seem to offer a new scale of precision and personalisation, Commissioners were concerned about the extent to which young people could be properly listened to, and consulted with, via automated real-time monitoring and feedback.

*ii) Digital Platforms + AI = AI-Enabled Communication*

WH&Y Commissioners reported feelings of resignation and compliance about the scale and reach of ubiquitous *digital platforms*. For instance, young people are aware of the business models of digital platforms and data, driven by increasingly sophisticated marketing and advertising. They expressed feelings of resignation that the monetisation of personal data seems unlikely to change, especially because of the convenience and inevitability of information sharing. A sense of compliance to the current digital system of Terms and Conditions was also raised, by way of feeling forced to agree even if one does not agree with how the system operates by extracting data. Commissioners also reported the potential of collaboration with, and between, platforms as a way to negotiate change (for example corporations working with young people to design platforms). The production lifecycle of the technology devices which support ubiquitous access to digital platforms also drew attention to environmental costs, such as e-waste contributing to landfill. Commissioners discussed *artificial intelligence (AI*) chatbots as an illustrative example of AI's novel data collection and use. There was a view that chatbots lack emotion and empathy, which might fail to recognise young people's diverse needs, and that there was no opportunity for building relationships with health professionals, or service providers. They could not make a reciprocal connection with a chatbot, and that chatbot interactions are primarily transactional. Furthermore, the chatbot was viewed as potentially taking away or even de-valuing, personal interactions. Commissioners reported on the ambiguities, or grey areas, associated with the innovative practice of *AI-enabled communication* - especially understanding whether the primary motivation for the roll-out of AI is cost-savings, or care. Concerns identified about AI-enabled communication included whether interactions with chatbots could possibly be demotivating for a young person because they might not feel heard, or become less proactive by engendering a false sense of time spent on health and well-being. Being nudged towards healthy behaviour could also be ignored over time, or young people could lie about their activities. Nudging could also exacerbate ‘Dr Google’ syndrome: the feeling of not knowing if something is serious, and pathologising health concerns. Another strong view was that sometimes things change for the sake of it, rather than to address persistent challenges. For example, a new technology will not simply resolve certain issues in some culturally and linguistically diverse communities where knowledge and trust about existing health services is a key issue.

*iii) Data Records + Big Data = Data-Intensive Research*

Commissioners discussed that ubiquitous *data records* (both digitised clinical data collected by health professionals, and data collected by other organisations) could potentially exacerbate issues around inequality and surveillance now and in the future. They felt a more intense focus on data collection obligates young people to become more diligent about recording their information to be seen by decision-makers, or subjects them to new levels of surveillance by family, peers, corporations, and service-providers. Subsequently, certain groups of young people may become less visible to organisations than others, especially those who cannot afford, or easily access technology. Another concern was the biases introduced when certain population groups of young people were under-represented or over-represented in database records. This could lead to research, policy, and practice being influenced by over-represented groups. Commissioners’ view of *big data* corresponded to secondary use of data as an illustrative example of novel data collection and use associated with big data. They suggested that making big data processes more visible and understandable could encourage young people to make more informed choices and decisions about their data especially signing up for services or sharing information. Better understanding where their data were going, plus transparency about how big data processes operate, would help engender trust in technological use and application. In terms of the innovative practice of *data-intensive research*, Commissioners expressed the need for having greater control over their data - especially as their activities are being monitored and used for multiple purposes. Feelings of lack of control were associated with the view that data were difficult to delete, especially if shared with many different professionals and organisations. Being able to self-regulate what data are given to others via devices and platforms was viewed positively, as was the idea that the proceeds of data should be for public benefit and good across health and other domains.

## Discussion

Within this study, concepts associated with emerging technologies were clarified by developing a *Digital Innovation Typology* from a theory building review and utilised to support two-way learning between researchers and young people in our adolescent health research community of practice. Our typology advances research in a relatively new field by providing a systemic, flexible and adaptable tool for youth and adult stakeholders to navigate and negotiate the emerging technologies and interrelationships that shape health systems innovation. A typology has been developed to navigate the uncertainty associated with digital public health technologies during COVID-19,^66^ demonstrating the utility of such navigation aids for policymakers and decision-makers to help guide the ethical development of tools. Also, a systematic review of eHealth implementation typologies identified that existing theories focus on individual readiness, rather than social or systemic aspects of implementation,^67^ supporting the novelty of our typology.

Nine key insights were identified from a synthesis of review and workshop findings.

1. The ubiquitous access of *networked devices* can conveniently support young people's health-related decision-making with digital nudges, yet negative pressures and privacy issues associated with datafication must be investigated. The datafication of health means that data about health can be generated ubiquitously, is increasingly personalised, and is used to make profiles, predictions, and scores about people's health and well-being. The COVID-19 pandemic is accelerating data generation even further, with the transition of many government, economic, social and cultural systems to digital services.^68^
2. The installed base and imposed standards of dominant *digital platforms* means that technology use is increasingly reliant upon corporate growth business models and data brokers. Manufacturers’ control of products and services and restricted repairs is an important consideration, as the health sector becomes increasingly corporatized, commercialised, and monitored by data brokers.^69^ The Right to Repair movement reflects growing community concerns about existing processes of technology repair, waste and planned obsolescence, such as ‘exclusionary design tactics’ based on highly specialized, noninterchangeable parts increase costs.^70,71^ Market conditions discriminate against young people who rely on their parent/guardian or educational institution for access to technology. Affordable broadband access alongside investment in digital skills and abilities for digitally excluded populations are vital for COVID-19 recovery initiatives.^72^
3. Expanding *data records* are increasingly embedded across society and leveraged for health system decision-making, so the potential for inequities associated with young people's digital inclusion/exclusion should be addressed. In an increasingly datafied economy, the prevalent trend is extraction and exploitation of young people's data, instead of supporting their agency and well-being.^73^ An integrated and comprehensive approach to data governance is vital to address disparate impacts on vulnerable, low-income, and culturally diverse population groups of young people.^74^
4. As *IoT* expands the reach and scope of data collection and use across health systems, young people's concerns about data mis-representation and manipulation deserve closer scrutiny. IoT is viewed as central to health systems innovation across clinical and consumer contexts. The integration of IoT to diagnose, monitor and treat patients is being built into a range of health-related products and services, requiring new forms of privacy discussions and digital literacy initiatives with consumers and clinicians.^75,76^
5. The spread of *AI* has the potential to both limit and expand young people's relationships with health professionals and service providers. Policy guidance on AI for young people recommends: support for development and well-being; prioritising fairness and non-discrimination; protecting their data and privacy; ensuring inclusion and safety; providing transparency explicability, and accountability; preparing them for present and future AI developments; empowering governments and businesses with knowledge of AI and children's rights; and creating and enabling environments for child-centred AI.^77^
6. Increased visibility about the *big-data* production life-cycle is required to support young people's informed decision-making about novel data collection and use across health systems. The opportunities of biomedical big data include new forms of monitoring and prediction. Persistent challenges are ‘informed consent, privacy, confidentiality, diversity, data ownership, digital divides, collective rights, and inclusive governance of research data’^78^^, p.18^. The COVID-19 pandemic has amplified data-related challenges, and strengthened calls for the responsible use of digital data.^79^ Efforts to advance digital health governance are beginning to address unique impacts on children and young people.^80^
7. Young people believe that distributed health-related roles and conventions of practice are being tested with applications of *real-time monitoring*. Health-related IoT could lead to reduced face-to-face interactions, narrow healthcare to measurements and automated processes, undermine the relationship between patients and professionals, and shift responsibility regarding care to the individual, family, friends and community.^81^ Wearable patient monitoring systems face potential technical barriers, including unreliable or disturbed signals; delays in results and alerts; accuracy and reliability of decision-support systems.^82^
8. The integration of *AI-enabled communication* in diverse health system contexts should seek to balance the interests of youth and adult stakeholders. Systemic factors influencing AI implementation in healthcare include people (attitudes of professionals and the public, trust, work and training, deskilling and disempowerment); the health system (leadership, management, clinical responsibility, regulation); plus data and tool related issues: (privacy, consent, ownership, transparency, reliability, and bias.^83^
9. Health systems models of *data-intensive research* must be responsive to young people's needs and communities. Emergent models of big-data related research include non-profit/community-led projects (Open Humans), commercial projects (PatientsLikeMe) and cooperatives (MiData).^84^ Participants’ roles in this research vary according to the organisational approach, resources, and literacies of people involved: providers of biomaterials and data; administrators of their own research participation; (co-) principal investigators.^85,86^ While ethical practices and data governance principles, actions to support responsible and rights-based approaches to children and young people's data are on the rise,^87–89^ young people's views about big-data related research are underexplored.

Our study results offer a unique and flexible tool and vocabulary for integration across a range of future research and priority-settings agendas (Table 2). These insights are beneficial because young people and their data are deeply embedded, yet largely invisible, in the digital health system. Digital health technologies and their applications are not designed with young people in mind, further disempowering them and potentially compromising their health equity in the longer term.^90^ This study therefore provides a flexible foundation of resources to expand future health systems research with young people. Amid digital health uncertainty and varying contexts of use, this shared learning can assist in surfacing and shaping how technologies are designed, implemented, and used.^33^

**Table 2.** Implications for future research and priority-setting agendas.

1.	Explore young people's views about datafication, digital nudging and consent so as to design and develop networked devices across health systems innovation which support, rather than detract, from youth health and well-being.
2.	Examine young people's concerns about data, privacy, and the environmental impacts of technology to co-produce health systems innovation which prioritises their views about accessibility and sustainability.
3.	Explore young people's experiences and expectations regarding data records so that government, academic, and industry stakeholders learn from, and address, young people's concerns and ideas about how data records should be designed, maintained, and utilised.
4.	Investigate young people's views about the potential application of IoT across these diverse health-related contexts so that opportunities and challenges are fully addressed with adult stakeholders across health systems innovation.
5.	Examine how young people view the limits and possibilities of AI: for example, how chatbots can enable or constrain meaningful relationships with health professionals, plus inform the design and development of safe and secure adolescent health services.
6.	Communicate and visualise the complexity of big data with young people and be translated to diverse youth and adult stakeholders - so as to help build more meaningful and trustworthy health systems.
7.	Identify how young people view the social implications of real-time monitoring across health systems, the impact of associated technical barriers across different contexts (for example, urban, regional and remote areas), plus making solutions based on identified opportunities and challenges.
8.	Explore young people's views about AI-enabled communication in dialogue with adult stakeholders across the health system; this should include the choice to decline, or refuse, developments which don’t align with their shared views or values.
9.	Examine emergent models of data-intensive research, so young people's aspirations for control, agency, and contributing to the public good, are prioritised as digital transformation evolves.

This study has some limitations. As an integrated knowledge translation study, the invited participants were restricted to stakeholders within the WH&Y NHMRC Centre of Research Excellence in Adolescent Health. Reported findings must therefore be contextualised within this particular community of practice that seeks to integrate youth engagement across health research and translation in the Australian context. Future research could consider utilising the typology as a negotiation tool to shape future health systems research and priority-setting agendas with specific organisations and groups of young people beyond this research initiative. For example, young people in regional and remote areas, young people experiencing homelessness, or living in poverty. Another limitation was that the workshop with young people was conducted online, due to COVID-related restrictions at the time. Future research would benefit from in-person workshops which enable more interactive, hands-on activities and dialogue between participants. Beyond the Australian context, the *Digital Innovation Typology* could also be explored further in other country, cultural, organisational, and community contexts to support two-way learning about emerging technologies between youth and adult stakeholders (health professionals, interdisciplinary researchers, and intersectoral partners). Furthermore, both the typology and accompanying *Digital Health Dictionary* are designed to be systemic, shareable and adaptable resources to support ongoing learning as technologies converge and concepts evolve. For example, QR codes for COVID-19 contact tracing, virtual reality (VR) software for health-related VR, personal online data stores (Pods) for personal web-server hosting, or general-purpose artificial intelligence models (such as large language models, for instance ChatGPT).

## Conclusion

Our study findings support mutual learning between youth and adult stakeholders within, and beyond, our adolescent health community of practice: by making sense together about the evolving and complex technologies increasingly used in health systems innovation and everyday life in a rapidly changing society. We recommend that health systems change would benefit from moving away from individual, adult, or siloed organisation decision-makers — and towards *intergenerational* and *intersectoral* dialogue and action. The complexity of health systems can only be understood, interpreted, tested, decided, and acted upon in this integrative way: together, in systemic ways, and over time.
